# Evaluating cardiac disorders associated with triazole antifungal agents based on the US Food and Drug Administration Adverse Event reporting system database

**DOI:** 10.3389/fphar.2024.1255918

**Published:** 2024-03-20

**Authors:** Jinhua Chen, Shijun Xu, Weijiang Yu, Cuicui Sun, Wenzhou Zhang

**Affiliations:** ^1^ Department of Pharmacy, The Affiliated Cancer Hospital of Zhengzhou University and Henan Cancer Hospital, Henan Engineering Research Center for Tumor Precision Medicine and Comprehensive Evaluation, Henan Provincial Key Laboratory of Anticancer Drug Research, Zhengzhou, China; ^2^ Department of Interventional Radiology, The Affiliated Cancer Hospital of Zhengzhou University and Henan Cancer Hospital, Zhengzhou, China; ^3^ Department of Pharmacy, Qilu Hospital of Shandong University, Ji’nan, China

**Keywords:** triazole antifungal agents, FAERS, cardiac disorders, adverse events, isavuconazole

## Abstract

**Introduction:**

Triazole antifungal agents are widely used to treat and prevent systemic mycoses. With wide clinical use, the number of reported adverse events has gradually increased. The aim of this study was to analyze the cardiac disorders associated with TAAs (fluconazole, voriconazole, itraconazole, posaconazole and isavuconazole) based on data from the US Food and Drug Administration Adverse Event Reporting System FDA Adverse Event Reporting System.

**Methods:**

Data were extracted from the FAERS database between the first quarter of 2004 and third quarter of 2022. The clinical characteristics in TAA-associated cardiac AE reports were analyzed. Disproportionality analysis was performed to evaluate the potential association between AEs and TAAs using the reporting odds ratio (ROR) and proportional reporting ratio (PRR).

**Results:**

Among 10,178,522 AE reports, 1719 reports were TAA-associated cardiac AEs as primary suspect drug. Most reports were related to fluconazole (38.34%), voriconazole (28.56%) and itraconazole (26.76%). Itraconazole (N = 195, 42.39%) and isavuconazole (N = 2, 14.29%) had fewer serious outcome events than three other drugs including fluconazole, voriconazole, and posaconazole. 13, 11, 26, 5 and 1 signals were detected for fluconazole, voriconazole, itraconazole, posaconazole and isavuconazole, respectively. The number of new signals unrecorded in the drug label was 9, 2, 13, 2 and 0 for fluconazole, voriconazole, itraconazole, posaconazole and isavuconazole, respectively.

**Conclusion:**

Isavuconazole might be the safest of the five TAAs for cardiac AEs. TAA-associated cardiac disorders may result in serious adverse outcomes. Therefore, in addition to AEs on the drug label, we should pay attention to new AEs unrecorded on the drug label during the clinical use of TAAs.

## 1 Introduction

Triazole antifungal agents (TAAs) are used for front-line prophylaxis and as therapy for many systemic mycoses owing to their broad antifungal spectrum and low toxicity (Khanina et al., 2021; Stemler et al., 2023). They inhibit the cytochrome P450 (CYP)-dependent 14-а-demethylase to prevent the synthesis of ergosterol from lanosterol in the fungal cell membrane, thereby causing fungal cell death (Lass-Flörl, 2011). Clinically available TAAs include fluconazole, voriconazole, itraconazole, posaconazole and isavuconazole. TAAs have different recommendations in clinical practice based on their different antibacterial spectra, pharmacokinetics and the severity of the disease. The most common systemic mycoses in clinic are caused by *Candida* and *Aspergillus* infections (Ikuta et al., 2024). Fluconazole has good antifungal activity against *Candida* (except for *Candida krusei* and *Candida glabrata*), but no activity against *Aspergillus*. It is generally recommended for the treatment of mild *Candida*-infected patients without a history of prophylactic use of azole antifungal agents (Chinese Association Hematologists, 2020). Voriconazole, itraconazole, posaconazole and isavuconazole all have good antifungal activity against *Candida* and *Aspergillus*, which are used to treat patients with *Candida* and/or *Aspergillus* infection. Voriconazole is recommended as the preferred drug to treat patients with invasive aspergillosism (Patterson et al., 2016; Ullmann et al., 2018). Moreover, mucormycosis is prone to occur in immunocompromised patients, and once it occurs, the mortality rate is high. Due to the outbreak of Coronavirus disease (COVID-19), the occurrence of mucormycosis has significantly increased (Muthu et al., 2024; Sharma et al., 2024). Currently, the only effective drugs for the treatment of mucormycosis are amphotericin B, posaconazole and isavuconazole (Cornely et al., 2019).

Many adverse events (AEs) have been reported in their post-marketing application, mainly including hepatotoxicity, gastrointestinal reaction, renal impairment, rash, and cardiac disorders (Amsden and Gubbins, 2017; Shen et al., 2022; Yu and Liao, 2022; Zhou et al., 2022). Among these, cardiac disorders were a significant systemic organ class (SOC) signals for important medical events (IMEs) induced by TAAs (Zhou et al., 2022). Cardiac disorders are common clinical conditions associated with high mortality rates (Shinomoto et al., 2022). There are many manifestations such as torsade de pointes, long qt syndrome, ventricular tachycardia and cardiac failure (Perpinia et al., 2022). Many possible mechanisms are related to the occurrence of TAA-induced cardiac disorders. Various factors caused the increased concentration of TAAs to lead to the occurrence of cardiac disorders, such as the variation of cytochrome P450 (CYP) activity or polymorphisms, and overdosage (Amsden and Gubbins, 2017; Teaford et al., 2020). Moreover, TAAs were observed to change the concentration of other drugs caused by drug-drug interactions, which led to the occurrence of cardiac disorders (Mourad et al., 2019; Yuan et al., 2023).

There have been many reports about TAA-induced cardiac disorders in recent years. It included arrhythmia, bradycardia, cardiac arrest, qt interval prolongation, torsade de pointes et al. (Salem et al., 2017; Mellinghoff et al., 2018; Zhou et al., 2022; Yuan et al., 2023). Among these, qt interval prolongation was mainly found in fluconazole, voriconazole, itraconazole, and posaconazole (Yu and Liao, 2022). In addition to these cardiac AEs on the drug label, some new serious cardiac AEs unrecorded on the drug label are gradually discovered with the increase of their clinical use. For example, itraconazole induced heart failure when it was administrated at the dosage of 200 mg twice a day or once a day (Paleiron et al., 2011; Abraham and Panda, 2018). Isavuconazole shortened the qt interval according to the drug label, however, it was demonstrated that isavuconazole could induce qt interval prolongation at regular dosage as specified in the drug label in recent years (Marty et al., 2016; Zhou et al., 2022). Thus, it is of important significance to explore new cardiac AEs to ensure drug safety.

To the best of our knowledge, there are currently several pharmacovigilance systems established by countries or organizations, such as the World Health Organization, China National Medical Products Administration, US Food and Drug Administration (FDA), and European Medicines Agency, to monitor adverse reactions. Of these, the FDA Adverse Event Reporting System (FAERS) is the largest public database for the monitoring of drugs and therapeutic biologics in post-marketing use (Rodriguez et al., 2001; Banda et al., 2016). It includes AEs caused by all FDA-approved drugs (Sakaeda et al., 2013). However, there have been no comprehensive reports on cardiac disorders regarding TAAs. In this study, we comprehensively evaluated TAA-induced cardiac disorders by a disproportionality analysis using the FAERS, aiming to that it can serve a better clinical application for different populations.

## 2 Materials and methods

### 2.1 Data sources and processing

The FAERS database is a public database of self-reported AEs in many countries worldwide. Data from the FAERS database are released quarterly. We obtained TAA-associated cardiac AEs from the first quarter of 2004 to third quarter of 2022 from the FAERS database using OpenVigil 2.1 tool, and conducted a pharmacovigilance study using these data (Böhm et al., 2021). In this study, TAAs included fluconazole, voriconazole, itraconazole, posaconazole and isavuconazole. AE names were coded based on preferred term (PT) codes and SOCs using the Medical Dictionary for Regulatory Activities (MedDRA, version 25.1).

**FIGURE 1:**
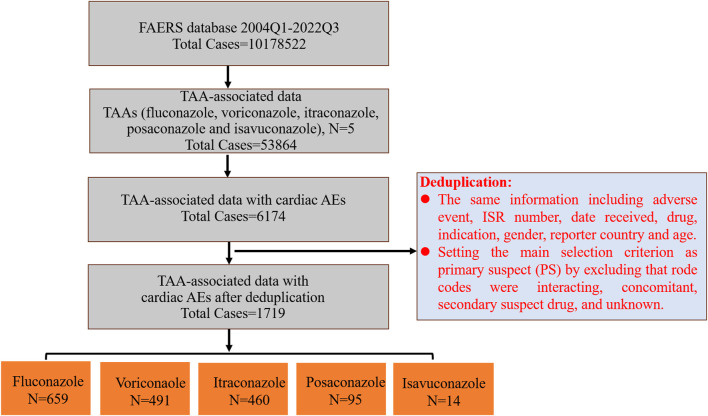
The flow diagram of screening TAA-associated cardiac AEs from the FAERS database.

Then we further screened and deduplicated these data. Firstly, all PTs associated with each TAA were extracted from the first quarter of 2004 to third quarter of 2022 from the FAERS database, and were grouped into different SOCs. Secondly, PTs from the SOC coded as “cardiac disorders” were selected out and used for the subsequent analysis. Thirdly, cardiac AEs associated with each TAA were extracted based on these enrolled PTs. Lastly, we deduplicated the reports of TAA-associated cardiac AEs, and the detailed screening process was shown in Figure 1. Reports with the same information including adverse event, ISR number, date received, drug, indication, gender, reporter country and age were identified as duplicate reports and excluded. Then we further selected out AEs as primary suspect (PS) by excluding in which AEs may have occurred due to interacting drugs, concomitant drugs, secondary suspect drugs, and other unknown. After the above deduplication, the remaining reports were used for follow-up analysis.

Clinical characteristics in enrolled reports with TAA-associated cardiac AEs were analyzed, including sex, age, reporting region, outcome and reporting year. Serious outcomes included hospitalization, life-threatening, disability, congenital anomaly, and death.

### 2.2 Signal mining

In this study, the disproportionality analysis was performed to evaluate the potential association between AEs and TAAs using the reporting odds ratio (ROR) and proportional reporting ratio (PRR) (Alkabbani and Gamble, 2021; Greten et al., 2021). Cardiac AEs were considered to be positive and associated with the corresponding drugs when the ROR and PRR both met the criteria (Supplementary Table S1).

### 2.3 Statistical analysis

Descriptive analyses were performed to summarize the clinical characteristics in the reports of TAA-associated cardiac AEs. All data mining and statistical analyses were performed using Microsoft Excel 2019 and Microsoft PowerPoint 2019 (Microsoft, Redmond, Washington, United States).

## 3 Results

### 3.1 Descriptive analyses

There were totally 10,178,522 AEs in the FAERS database from 2004 Q1 to 2022 Q3. Among these, 53,864 (0.53%) AEs were attributed to TAAs, including fluconazole, voriconazole, itraconazole, posaconazole and isavuconazole, of which 6,174 were cardiac AEs (11.46%). Voriconazole (39.71%) and fluconazole (35.19%) accounted for the highest percentage of TAA-associated AEs, followed by itraconazole (12.96%), posaconazole (7.32%) and isavuconazole (4.82%); However, fluconazole accounted for the highest percentage of TAA-associated cardiac AEs (42.65%), followed by voriconazole (32.09%), itraconazole (19.48%), posaconazole (5.39%), and isavuconazole (0.39%) (Table 1). Further screening about TAA-associated cardiac AEs as PS was performed by excluding “Rode code” as interacting, concomitant, secondary suspect drug, and unknown. There were totally 1719 reports with TAAs as PS drugs. The orders by proportion from highest to lowest were fluconazole (38.34%), voriconazole (28.56%), itraconazole (26.76%), posaconazole (5.53%), and isavuconazole (0.81%), and the orders were the same as that of TAA-associated cardiac AEs.

**TABLE 1:** Distribution of TAA-associated AEs and cardiac AEs.

Drug name	TAA-associated AEs n (%)	TAA-associated cardiac AEs n (%)	TAA-associated cardiac AEs as PS n (%)
Fluconazole	18,954 (35.19)	2,633 (42.65)	659 (38.34)
Voriconazole	21,391 (39.71)	1981 (32.09)	491 (28.56)
Itraconazole	6,982 (12.96)	1203 (19.48)	460 (26.76)
Posaconazole	3943 (7.32)	333 (5.39)	95 (5.53)
Isavuconazole	2,594 (4.82)	24 (0.39)	14 (0.81)
Total	53,864	6,174	1719

Table 2 describes the clinical characteristics in reports of TAA-associated cardiac AEs. The mean age in the reports was 55.24 years. There were similar reported numbers of males and females (45.67% vs. 41.42%). These reports were mainly from the United States (32.98%), followed by Japan (10.82%) and China (9.19%). Hospitalization was the most frequent serious outcome event (22.80%), followed by death (16.11%), and life-threatening (13.96%). Fluconazole (N = 407, 61.76%) resulted in the most serious outcome events, followed by voriconazole (N = 277, 56.42%), posaconazole (N = 65, 68.42%), itraconazole (N = 195, 42.39%), and isavuconazole (N = 2, 14.29%). Moreover, 5%–8% of reports in 2015–2022 were related to cardiac AEs, and around 3%–5% were related to cardiac AEs in other years (Table 3).

**TABLE 2:** Clinical characteristics in reports of TAA-associated cardiac AEs.

	Fluconazole	Voriconazole	Posaconazole	Itraconazole	Isavuconazole	Total (%)
Age, years						
Mean	51.40	60.07	49.36	56.86	58.50	55.24
Not reported	147	126	20	138	6	437
Gender						
Male	234	264	51	231	5	785 (45.67)
Female	344	156	38	165	9	712 (41.42)
Not reported	81	71	6	64	0	222 (12.91)
Reporting region						
United States	226	188	21	120	12	567 (32.98)
Japan	24	76	1	85		186 (10.82)
China	38	54	5	61		158 (9.19)
France	36	42	11	19	1	109 (6.34)
United Kingdom	55	7	17	52		131 (7.62)
Germany	44	2	9	8		63 (3.66)
Canada	24	5	1	4		34 (1.98)
Other countries	212	117	30	111	1	471 (27.40)
Outcomes						
Hospitalization	160	93	31	107	1	392 (22.80)
Death	77	128	23	48	1	277 (16.11)
Life-Threatening	140	54	10	36		240 (13.96)
Disability	15	2	1	4		22 (1.28)
Congenital Anomaly	15					15 (0.87)
Others and unknown	252	214	30	265	12	773 (44.97)

**TABLE 3:** The counts of patients with TAA-associated cardiac AEs yearly from 2004 Q1 to 2022 Q3.

	Fluconazole	Voriconazole	Posaconazole	Itraconazole	Isavuconazole	Total (%)
2004	24	26		28		78 (4.54%)
2005	23	21		19		63 (3.66%)
2006	31	19	1	14		65 (3.78%)
2007	22	11	7	20		60 (3.49%)
2008	42	4	4	34		84 (4.89%)
2009	17	16	5	31		69 (4.01%)
2010	20	32	1	24		77 (4.48%)
2011	31	31	6	24		92 (5.35%)
2012	34	38	6	30		108 (6.28%)
2013	31	22		17		70 (4.07%)
2014	24	20	2	15		61 (3.55%)
2015	45	29	5	12	1	92 (5.35%)
2016	61	28	6	11	1	107 (6.22%)
2017	57	30	11	20	3	121 (7.04%)
2018	56	30	14	37	1	138 (8.03%)
2019	49	41	6	24	2	122 (7.10%)
2020	31	30	6	68	1	136 (7.91%)
2021	28	26	10	21	2	87 (5.06%)
2022	33	37	5	11	3	89 (5.18%)

Daily dosage distribution of these five TAAs were further analyzed. There were 250 reports that the dosage of fluconazole was known, followed by 246 reports for voriconazole, 93 reports for itraconazole, 43 reports for posaconazole, and 2 reports for isavuconazole. According to the instructions, the routine daily dosage of fluconazole, voriconazole, posaconazole, itraconazole, and isavuconazole are 400, 400, 300, 200, and 200 mg, respectively. For these reports of known dosage, it showed that 235 reports (235/250, 94.00%), 215 reports (215/246, 87.40%), 37 reports (37/93, 39.78%), 24 reports (24/43, 55.81%), and one report (1/2, 50.00%) did not exceed the routine daily dosage for fluconazole, voriconazole, itraconazole, posaconazole and isavuconazole, respectively (Supplementary Table S2).

### 3.2 Disproportionality analyses

We screened for positive TAA-associated cardiac adverse signals based on the criteria for ROR and PRR. Fluconazole, voriconazole, itraconazole, posaconazole, and isavuconazole produced 13, 11, 26, 5 and one signals, respectively (Table 4). Many new cardiac AEs that were unrecorded on the drug label were found in our data mining from FAERS, including transposition of the great vessels, Kounis syndrome, Fallot’s tetralogy, ventricular fibrillation, atrioventricular block first degree, ventricular extrasystoles, sinus bradycardia, pericarditis and cardiac failure chronic for fluconazole; ventricular hypokinesia and pericardial effusion for voriconazole; reperfusion arrhythmia, atrioventricular block, left ventricular dysfunction, torsade de pointes, supraventricular extrasystoles, ventricular extrasystoles, myocarditis, cardiomegaly, haemoptysis, pericardial effusion, atrioventricular block complete, atrial flutter and arteriosclerosis coronary artery for itraconazole; and cardiac arrest for posaconazole. No new cardiac AEs were associated with isavuconazole.

**TABLE 4:** Signal strength of TAA-associated cardiac AEs at the PT level in the FAERS database.

TAAs	Preferred term (PT)	Report number	ROR (95%CI)	PRR (95%CI)
Fluconazole	Long qt syndrome	31	51.58 (36.06–73.78)	51.30 (35.93–73.24)
	Torsade de pointes	87	40.57 (32.75–50.25)	39.96 (32.36–49.34)
	Transposition of the great vessels	11	37.94 (20.87–68.98)	37.87 (20.85–68.77)
	Kounis syndrome	10	21.14 (11.33–39.46)	21.11 (11.32–39.35)
	Fallot’s tetralogy	6	15.49 (6.93–34.62)	15.48 (6.93–34.55)
	Ventricular tachycardia	38	9.29 (6.75–12.79)	9.24 (6.72–12.69)
	Ventricular arrhythmia	8	8.17 (4.08–16.36)	8.16 (4.07–16.33)
	Ventricular fibrillation	21	7.46 (4.86–11.46)	7.44 (4.85–11.41)
	Atrioventricular block first degree	5	4.15 (1.73–9.99)	4.15 (1.73–9.97)
	Ventricular extrasystoles	9	3.59 (1.86–6.90)	3.58 (1.86–6.89)
	Sinus bradycardia	8	3.34 (1.67–6.69)	3.59 (1.09–10.52)
	Pericarditis	7	3.10 (1.48–6.52)	3.34 (1.67–6.68)
	Cardiac failure chronic	3	3.39 (1.09–10.53)	3.10 (1.48–6.50)
Voriconazole	Fungal endocarditis	9	129.64 (65.55–256.39)	129.47 (65.52–255.85)
	Torsade de pointes	21	7.75 (5.04–11.91)	7.73 (5.04–11.86)
	Sinus arrest	3	6.45 (2.07–20.05)	6.45 (2.07–20.03)
	Haemoptysis	41	5.36 (3.94–7.30)	5.34 (3.93–7.25)
	Ventricular hypokinesia	4	4.30 (1.61–11.47)	4.30 (1.61–11.46)
	Cardiotoxicity	8	3.11 (1.55–6.23)	3.11 (1.55–6.22)
	Cardiac failure acute	5	3.10 (1.29–7.46)	3.10 (1.29–7.45)
	Ventricular tachycardia	12	2.37 (1.35–4.18)	2.37 (1.35–4.17)
	Supraventricular tachycardia	7	2.45 (1.17–5.14)	2.45 (1.17–5.14)
	Pericardial effusion	14	2.23 (1.32–3.76)	2.22 (1.32–3.76)
	Ventricular extrasystoles	7	2.27 (1.08–4.77)	2.27 (1.08–4.76)
Itraconazole	Reperfusion arrhythmia	3	417.37 (126.54–1376.60)	416.90 (126.55–1373.45)
	Cardiotoxicity	49	50.09 (37.69–66.56)	49.20 (37.21–65.05)
	Acute left ventricular failure	4	49.93 (18.61–134.01)	49.86 (18.61–133.63)
	Left ventricular failure	6	16.61 (7.44–37.08)	16.58 (7.44–36.93)
	Atrioventricular block	14	14.82 (8.76–25.09)	14.75 (8.74–24.90)
	Left ventricular dysfunction	9	13.27 (6.89–25.57)	13.23 (6.88–25.43)
	Torsade de pointes	13	12.27 (7.11–21.17)	12.21 (7.09–21.02)
	Supraventricular extrasystoles	4	10.87 (4.07–29.02)	10.85 (4.07–28.94)
	Cardiac failure acute	6	9.53 (4.27–21.25)	9.51 (4.27–21.17)
	Ventricular extrasystoles	9	7.49 (3.89–14.41)	7.46 (3.89–14.34)
	Myocarditis	7	6.81 (3.24–14.31)	6.80 (3.24–14.25)
	Cardiac failure	55	6.12 (4.68–7.99)	6.01 (4.63–7.81)
	Cardiomegaly	9	6.11 (3.18–11.77)	6.10 (3.17–11.71)
	Haemoptysis	18	6.01 (3.78–9.56)	5.98 (3.77–9.48)
	Pericardial effusion	14	5.71 (3.38–9.66)	5.69 (3.37–9.60)
	Sinus bradycardia	6	2.29 (2.34–11.65)	5.21 (2.34–11.60)
	Pulmonary oedema	22	4.53 (2.97–6.89)	4.50 (2.97–6.82)
	Atrioventricular block complete	4	5.08 (1.91–13.56)	5.08 (1.91–13.53)
	Arrhythmia	27	4.43 (3.03–6.48)	4.40 (3.02–6.40)
	Oedema peripheral	59	3.95 (3.05–5.11)	3.88 (3.02–5.00)
	Atrial flutter	4	4.50 (1.69–12.00)	4.49 (1.69–11.97)
	Arteriosclerosis coronary artery	3	3.82 (1.23–11.86)	3.82 (1.23–11.83)
	Right ventricular failure	3	3.58 (1.15–11.10)	3.57 (1.15–11.08)
	Cardiac failure congestive	26	2.21 (1.50–3.25)	2.20 (1.50–3.22)
	Bradycardia	15	2.29 (1.38–3.81)	2.29 (1.38–3.79)
	Chest discomfort	23	2.18 (1.45–3.28)	2.17 (1.44–3.26)
Posaconazole	Ventricular arrhythmia	3	11.22 (3.61–34.82)	11.19 (3.61–34.68)
	Torsade de pointes	4	6.61 (2.48–17.65)	6.60 (2.48–17.57)
	Ventricular tachycardia	6	5.34 (2.40–11.92)	5.33 (2.40–11.84)
	Cardiac arrest	14	2.44 (1.44–4.14)	2.43 (1.44–4.09)
	Bradycardia	8	2.15 (1.07–4.31)	2.15 (1.08–4.28)
Isavuconazole	Haemoptysis	3	6.76 (2.17–21.05)	6.72 (2.18–20.74)

## 4 Discussion

In this study, we comprehensively evaluated cardiac disorders associated with TAAs in a real-world pharmacovigilance study of post-marketing drugs based on the FAERS database. In total, 1719 reports of cardiac AEs associated with TAAs as PS drugs were obtained after deduplication, accounting for 27.84% (1719/6174) of all AEs associated with TAAs, indicating that it is common and needs attention. According to the clinical characteristics in reports of TAA-related cardiac AEs, TAAs induce several serious outcome events including death, life-threatening, hospitalization, disability and congenital anomaly, especially fluconazole, voriconazole, posaconazole, and itraconazole. Isavuconazole was only reported in 14 cardiac AE reports, which mainly focused on other outcome events. Moreover, this study analyzed the yearly distribution of counts of patients from 2004 Q1 to 2022 Q3. The low number of isavuconazole-related cardiac AE reports may be attributed to the limited clinical application, which was approved in 2015.

Moreover, the occurrence of drug-induced AEs is usually related to its dosage. Daily dosage distribution analysis showed that some reports of drug dosage were unknown because the FAERS database is a self-reporting system and exists some missing data. For these reports of known dosage, fluconazole (94.00%) and voriconazole (87.40%) had large proportion of reports using no more than the routine daily dosage, indicating that it might be easy to be prone to cardiac AEs even if they were administrated no more than routine daily dosage. However, itraconazole and posaconazole only had 39.78% and 55.81% reports using no more than the routine daily dosage, indicating that the cardiac AEs they caused might be related to overdose use.

Based on TAA-associated adverse signals mining from the FAERS database, we screened their cardiac adverse signals and further obtained positive signals by the disproportionality analysis. To improve the sensitivity, specificity and predictive value, we selected two measures of disproportionality (ROR and PRR methods) together for signal detection. ROR compares the odds of reporting an event of interest for a particular drug to all other events, relative to the reporting odds for other drugs in the FAERS database; PRR is the proportion of spontaneous reports for a particular drug that are related to a particular adverse event, divided by the corresponding proportion for other drugs in the FAERS database (Rothman et al., 2004). van Puijenbroek et al. conducted a comparison of measures of disproportionality for signal detection in spontaneous reporting systems for adverse drug reactions, and demonstrated that there was no important difference between the measures. However, it was pointed out that each method has its advantages and disadvantages. The advantages of ROR method include easy applicable, different adjustments possible in logistic regression analysis, and interaction terms that can be used for the analysis of drug interactions and syndromes in logistic regression analysis, but it exists some disadvantages such as interpretation difficult, and occasionally impossible calculation when b or c is zero in its calculation formula. Nevertheless, PRR was easy interpretation and can still be calculated when c is zero (van Puijenbroek et al., 2002). Therefore, ROR and PRR can complement each other to some extent. The higher ROR or PRR value, the stronger association between the drug and the signal.

Based on the two disproportionality analysis methods, the results showed that itraconazole had the most PTs (*n* = 26), and 13 new cardiac PTs were unrecorded on the drug label, namely, reperfusion arrhythmia, atrioventricular block, left ventricular dysfunction, torsade de pointes, supraventricular extrasystoles, ventricular extrasystoles, myocarditis, cardiomegaly, haemoptysis, pericardial effusion, atrioventricular block complete, atrial flutter and arteriosclerosis coronary artery. Torsade de pointes are a type of ventricular tachycardia related to qt interval prolongation, which leads to sudden cardiac death (Uvelin et al., 2017; El-Sherif et al., 2018). Some epidemiological risk factors for this disease include gender, qt-prolonging drugs, ischemia and electrolyte imbalance (Sauer and Newton-Cheh, 2012). Torsade de pointes caused by itraconazole was mainly attributed to two reasons. One was the change of drug-metabolising enzymes associated with the clearance of itraconazole (Owens and Nolin, 2006), and the other was concomitant treatment with other drugs that can cause QT interval prolongation and torsade de pointes (Sagir et al., 2003; NoorZurani et al., 2009; Schwartz and Woosley, 2016). Moreover, cardiomegaly and pericardial effusion caused by itraconazole have been reported in the literature when the dosage, 400 mg/day, was administrated, but the mechanisms are not understood (Sasaki et al., 1999; Teaford et al., 2020). Therefore, we need to pay more attention to cardiac PTs, especially these new PTs, when itraconazole was used to treat. There were eight new cardiac PTs-related with fluconazole, including transposition of the great vessels, Kounis syndrome, Fallot’s tetralogy, atrioventricular block first degree, ventricular extrasystoles, sinus bradycardia, pericarditis, and cardiac failure chronic. Kounis syndrome, a hypersensitivity coronary syndrome or allergic angina, is divided into three types (Douedi et al., 2023). Fluconazole induced type 1 Kounis syndrome, and the mechanism was the same as other triggering entities, in which an allergen mediated IgE and mast cell activation and degranulation, causing the release of histamine (Singh Mahal, 2016). There were five cardiac PTs associated with posaconazole. Cardiac arrest induced by posaconazole is associated with long qt syndrome (Eiden et al., 2007; Panos et al., 2016). Moreover, there were only one cardiac PT associated with isavuconazole, and no new cardiac PTs in this study. A study conducted by Zhou et al. (2022) analyzed the distribution of AEs associated with TAAs in different SOCs from 2012 Q4 to 2022 Q1, and this analysis about the top 30 IMEs induced by TAAs based on all other drugs as analysis contexts showed that the number of the observed significant cardiac PT signals for fluconazole, voriconazole, itraconazole, posaconazole, and isavuconazole was 3, 4, 5, 4, and 1, respectively. Consistent with our results, it showed that isavuconazole had less cardiac PT signals compared to the other four TAAs. Therefore, it was speculated that isavuconazole might be the safest among the five TAAs. This may be partly due to its short time of clinical application. However, it needs to point out that these results were based on disproportionality analysis. It only shows the correlation statistically, but it did not reveal whether there was a causal relationship between adverse signals and drugs. Thus, it needs further observe and analyze with the increase of its clinical application.

Our study had a few limitations. First of all, the FAERS database is a self-reporting system and exists some flaws. For example, some significant data (e.g., sex and outcome) were lost. Secondly, only cases with AEs were reported to FAERS; thus, the incidence rate of each AE could not be assessed. Thirdly, disproportionality analysis based on the FAERS database statistically evaluated signals strength but it did not reveal whether there was a causal relationship between adverse signals and drugs. This needs to be confirmed by further clinical studies.

## 5 Conclusion

Our pharmacovigilance study mined and analyzed data on TAA-associated cardiac disorders in the FAERS database. These findings showed that TAA-associated cardiac disorders were common and drew attention, accounting for 27.84% of all AEs associated with TAAs. In addition to AEs on the drug label, some new AEs were unrecorded on the drug label, which may result in serious outcomes. Isavuconazole might be the safest of the five TAAs for cardiac AEs. However, further clinical studies are needed to elucidate the underlying causes and mechanisms.

## Data Availability

The original contributions presented in the study are included in the article/Supplementary Material, further inquiries can be directed to the corresponding author CS (cuicuisun1@126.com).

## References

[B1] Abraham A. O. Panda P. K. (2018). Itraconazole induced congestive heart failure, a case study. Curr. Drug. Saf. 13 (1), 59–61. 10.2174/1574886312666171003110753 28971777

[B2] Alkabbani W. Gamble J. M. (2021). Active-comparator restricted disproportionality analysis for pharmacovigilance signal detection studies of chronic disease medications: an example using sodium/glucose cotransporter 2 inhibitors. Br. J. Clin. Pharmacol. 89 (2), 431–439. 10.1111/bcp.15178 34964156

[B3] Amsden J. R. Gubbins P. O. (2017). Pharmacogenomics of triazole antifungal agents: implications for safety, tolerability and efficacy. Expert. Opin. Drug. Metab. Toxicol. 13 (11), 1135–1146. 10.1080/17425255.2017.1391213 29022838

[B4] Banda J. M. Evans L. Vanguri R. S. Tatonetti N. P. Ryan P. B. Shah N. H. (2016). A curated and standardized adverse drug event resource to accelerate drug safety research. Sci. Data. 3, 160026. 10.1038/sdata.2016.26 27193236 PMC4872271

[B5] Böhm R. Bulin C. Waetzig V. Cascorbi I. Klein H. J. Herdegen T. (2021). Pharmacovigilance-based drug repurposing: the search for inverse signals via OpenVigil identifies putative drugs against viral respiratory infections. Br. J. Clin. Pharmacol. 87 (11), 4421–4431. 10.1111/bcp.14868 33871897

[B6] Chinese Association Hematologists, Chinese Invasive Fungal Infection Working Group (2020). The Chinese guidelines for the diagnosis and treatment of invasive fungal disease in patients with hematological disorders and cancers (the 6th revision). Zhonghua. Nei. Ke. Za. Zhi. 59 (10), 754–763. 10.3760/cma.j.cn112138-20200627-00624 32987477

[B7] Cornely O. A. Alastruey-Izquierdo A. Arenz D. Chen S. C. A. Dannaoui E. Hochhegger B. (2019). Global guideline for the diagnosis and management of mucormycosis: an initiative of the European confederation of medical mycology in cooperation with the mycoses study group education and research consortium. Lancet. Infect. Dis. 19 (12), e405–e421. 10.1016/S1473-3099(19)30312-3 31699664 PMC8559573

[B8] Douedi S. Odak M. Mararenko A. Ross J. Sealove B. (2023). Kounis syndrome: a review of an uncommon cause of acute coronary syndrome. Cardiol. Rev. 31 (4), 230–232. 10.1097/CRD.0000000000000436 37335982

[B9] Eiden C. Peyrière H. Tichit R. Cociglio M. Amedro P. Blayac J. P. (2007). Inherited long QT syndrome revealed by antifungals drug-drug interaction. J. Clin. Pharm. Ther. 32 (3), 321–324. 10.1111/j.1365-2710.2007.00812.x 17489884

[B10] El-Sherif N. Turitto G. Boutjdir M. (2018). Acquired long QT syndrome and torsade de pointes. Pacing. Clin. Electrophysiol. 41 (4), 414–421. 10.1111/pace.13296 29405316

[B11] Greten S. Muller-Funogea J. I. Wegner F. Höglinger G. U. Simon N. Junius-Walker U. (2021). Drug safety profiles in geriatric patients with Parkinson's disease using the FORTA (Fit fOR the Aged) classification: results from a mono-centric retrospective analysis. J. Neural. Transm. (Vienna) 128 (1), 49–60. 10.1007/s00702-020-02276-x 33263172 PMC7815558

[B12] Ikuta K. S. Meštrović T. Naghavi M. (2024). Global incidence and mortality of severe fungal disease. Lancet. Infect. Dis. S1473-3099 (24), 00102–00106. 10.1016/S1473-3099(24)00102-6 38395046

[B13] Khanina A. Tio S. Y. Ananda-Rajah M. R. Kidd S. E. Williams E. Chee L. (2021). Consensus guidelines for antifungal stewardship, surveillance and infection prevention, 2021. Intern. Med. J. 51 (1), 18–36. 10.1111/imj.15586 34937134 PMC8206820

[B14] Lass-Flörl C. (2011). Triazole antifungal agents in invasive fungal infections: a comparative review. Drugs 71 (18), 2405–2419. 10.2165/11596540-000000000-00000 22141384

[B15] Marty F. M. Ostrosky-Zeichner L. Cornely O. A. Mullane K. M. Perfect J. R. Thompson G. R. 3rd. (2016). Isavuconazole treatment for mucormycosis: a single-arm open-label trial and case-control analysis. Lancet. Infect. Dis. 16 (7), 828–837. 10.1016/S1473-3099(16)00071-2 26969258

[B16] Mellinghoff S. C. Bassetti M. Dörfel D. Hagel S. Lehners N. Plis A. (2018). Isavuconazole shortens the QTc interval. Mycoses 61 (4), 256–260. 10.1111/myc.12731 29178247

[B17] Mourad A. Stiber J. A. Perfect J. R. Johnson M. D. (2019). Real-world implications of QT prolongation in patients receiving voriconazole and amiodarone. J. Antimicrob. Chemother. 74 (1), 228–233. 10.1093/jac/dky392 30295798

[B18] Muthu V. Agarwal R. Rudramurthy S. M. Thangaraju D. Shevkani M. R. Patel A. K. (2024). Risk factors, mortality, and predictors of survival in COVID-19-associated pulmonary mucormycosis: a multicentre retrospective study from India. Clin. Microbiol. Infect. 30 (3), 368–374. 10.1016/j.cmi.2023.12.006 38081413

[B19] NoorZurani M. H. Vicknasingam B. Narayanan S. (2009). Itraconazole-induced torsade de pointes in a patient receiving methadone substitution therapy. Drug. Alcohol. Rev. 28 (6), 688–690. 10.1111/j.1465-3362.2009.00128.x 19930027

[B20] Owens R. C. Jr Nolin T. D. (2006). Antimicrobial-associated QT interval prolongation: pointes of interest. Clin. Infect. Dis. 43, 1603–1611. 10.1086/508873 17109296

[B21] Paleiron N. Bizien N. Vinsonneau U. Andre M. Grassin F. (2011). Acute cardiac failure due to itraconazole. Rev. Mal. Respir. 28 (3), 352–354. 10.1016/j.rmr.2010.08.014 21482340

[B22] Panos G. Velissaris D. Karamouzos V. Matzaroglou C. Tylianakis M. (2016). Long QT syndrome leading to multiple cardiac arrests after posaconazole administration in an immune-compromised patient with sepsis: an unusual case report. Am. J. Case. Rep. 17, 295–300. 10.12659/ajcr.896946 27125217 PMC4913753

[B23] Patterson T. F. Thompson G. R. 3rd. Denning D. W. Fishman J. A. Hadley S. Herbrecht R. (2016). Practice guidelines for the diagnosis and management of aspergillosis: 2016 update by the infectious diseases society of America. Clin. Infect. Dis. 63 (4), e1–e60. 10.1093/cid/ciw326 27365388 PMC4967602

[B24] Perpinia A. S. Kadoglou N. Vardaka M. Gkortzolidis G. Karavidas A. Marinakis T. (2022). Pharmaceutical prevention and management of cardiotoxicity in hematological malignancies. Pharm. (Basel) 15 (8), 1007. 10.3390/ph15081007 PMC9412591 36015155

[B25] Rodriguez E. M. Staffa J. A. Graham D. J. (2001). The role of databases in drug post-marketing surveillance. Pharmacoepidemiol. Drug. Saf. 10 (5), 407–410. 10.1002/pds.615 11802586

[B26] Rothman K. J. Lanes S. Sacks S. T. (2004). The reporting odds ratio and its advantages over the proportional reporting ratio. Pharmacoepidemiol. Drug. Saf. 13 (8), 519–523. 10.1002/pds.1001 15317031

[B27] Sagir A. Schmitt M. Dilger K. Häussinger D. (2003). Inhibition of cytochrome P450 3A: relevant drug interactions in gastroenterology. Digestion 68 (1), 41–48. 10.1159/000073224 12949438

[B28] Sakaeda T. Tamon A. Kadoyama K. Okuno Y. (2013). Data mining of the public version of the FDA adverse event reporting system. Int. J. Med. Sci. 10, 796–803. 10.7150/ijms.6048 23794943 PMC3689877

[B29] Salem M. Reichlin T. Fasel D. Leuppi-Taegtmeyer A. (2017). Torsade de pointes and systemic azole antifungal agents: analysis of global spontaneous safety reports. Glob. Cardiol. Sci. Pract. 2017 (2), 11. 10.21542/gcsp.2017.11 29644223 PMC5871400

[B30] Sasaki E. Maesaki S. Kawamura S. Kakeya H. Ohno H. Hirakata Y. (1999). Itraconazole-induced hypokalemia in a patient with pulmonary aspergilloma. Nihon, Kokyuki, Gakkai, Zasshi. 37 (1), 36–40. 10087874

[B31] Sauer A. J. Newton-Cheh C. (2012). Clinical and genetic determinants of torsade de pointes risk. Circulation 125 (13), 1684–1694. 10.1161/CIRCULATIONAHA.111.080887 22474311 PMC3347483

[B32] Schwartz P. J. Woosley R. L. (2016). Predicting the unpredictable: drug-induced QT prolongation and Torsades de Pointes. J. Am. Coll. Cardiol. 67, 1639–1650. 10.1016/j.jacc.2015.12.063 27150690

[B33] Sharma A. Sharma A. Soubani A. O. (2024). Epidemiology of COVID 19-associated mucormycosis in the United States. Chest 165 (2), 307–312. 10.1016/j.chest.2023.09.012 37734565

[B34] Shen K. Gu Y. Wang Y. Lu Y. Ni Y. Zhong H. (2022). Therapeutic drug monitoring and safety evaluation of voriconazole in the treatment of pulmonary fungal diseases. Ther. Adv. Drug. Saf. 13, 20420986221127503. 10.1177/20420986221127503 36225945 PMC9549188

[B35] Shinomoto S. Tsubo Y. Marunaka Y. (2022). Detection and categorization of severe cardiac disorders based solely on heart period measurements. Sci. Rep. 12 (1), 17019. 10.1038/s41598-022-21260-x 36221030 PMC9553949

[B36] Singh Mahal H. (2016). Fluconazole-induced type 1 Kounis syndrome. Am. J. Ther. 23 (3), e961–e962. 10.1097/MJT.0000000000000113 26938747

[B37] Stemler J. Mellinghoff S. C. Khodamoradi Y. Sprute R. Classen A. Y. Zapke S. E. (2023). Primary prophylaxis of invasive fungal diseases in patients with haematological malignancies: 2022 update of the recommendations of the Infectious Diseases Working Party (AGIHO) of the German Society for Haematology and Medical Oncology (DGHO). J. Antimicrob. Chemother. 78, 1813–1826. 10.1093/jac/dkad143 37311136 PMC10393896

[B38] Teaford H. R. Abu Saleh O. M. Villarraga H. R. Enzler M. J. Rivera C. G. (2020). The many faces of itraconazole cardiac toxicity. Mayo. Clin. Proc. Innov. Qual. Outcomes. 4 (5), 588–594. 10.1016/j.mayocpiqo.2020.05.006 33083707 PMC7557188

[B39] Ullmann A. J. Aguado J. M. Arikan-Akdagli S. Denning D. W. Groll A. H. Lagrou K. (2018). Diagnosis and management of Aspergillus diseases: executive summary of the 2017 ESCMID-ECMM-ERS guideline. Clin. Microbiol. Infect. 24 (1), e1–e38. 10.1016/j.cmi.2018.01.002 29544767

[B40] Uvelin A. Pejaković J. Mijatović V. (2017). Acquired prolongation of QT interval as a risk factor for torsade de pointes ventricular tachycardia: a narrative review for the anesthesiologist and intensivist. J. Anesth. 31 (3), 413–423. 10.1007/s00540-017-2314-6 28229241

[B41] van Puijenbroek E. P. Bate A. Leufkens H. G. Lindquist M. Orre R. Egberts A. C. (2002). A comparison of measures of disproportionality for signal detection in spontaneous reporting systems for adverse drug reactions. Pharmacoepidemiol. Drug. Saf. 11 (1), 3–10. 10.1002/pds.668 11998548

[B42] Yu Z. Liao X. (2022). Torsade de Pointes/QT prolongation associated with antifungal triazoles: a pharmacovigilance study based on the U.S. FDA Adverse Event Reporting System (FAERS). J. Pharm. Pharm. Sci. 25, 237–243. 10.18433/jpps32867 35790147

[B43] Yuan Y. Wang C. Yao H. (2023). A case report of sudden cardiac arrest and torsade de pointes induced by the second-generation tyrosine kinase inhibitor dasatinib combined with fluconazole. Front. Cardiovasc. Med. 10, 984572. 10.3389/fcvm.2023.984572 36873392 PMC9975254

[B44] Zhou J. Wei Z. Xu B. Liu M. Xu R. Wu X. (2022). Pharmacovigilance of triazole antifungal agents: analysis of the FDA adverse event reporting system (FAERS) database. Front. Pharmacol. 13, 1039867. 10.3389/fphar.2022.1039867 36588707 PMC9798094

