# Erratum: Evaluating cardiac disorders associated with triazole antifungal agents based on the US food and drug administration adverse event reporting system database

**DOI:** 10.3389/fphar.2024.1425672

**Published:** 2024-05-16

**Authors:** Frontiers Production Office

**Affiliations:** Frontiers Media SA, Lausanne, Switzerland

**Keywords:** triazole antifungal agents, FAERS, cardiac disorders, adverse events, isavuconazole

Due to a production error, the equal contribution and shared first authorship of the authors Jinhua Chen and Shijun Xu was erroneously omitted. The following footnote has now been added to the authors Jinhua Chen and Shijun Xu:

† These authors have contributed equally to this work and share first authorship

The publisher apologizes for this mistake. The original version of this article has been updated.

