# Supplementary material for: Evaluating cardiac disorders associated with triazole antifungal agents based on the US Food and Drug Administration Adverse Event reporting system database
Source: Front Pharmacol. 2024 Mar 20;15:1255918. doi: 10.3389/fphar.2024.1255918 (PMC10997335; doi:10.3389/fphar.2024.1255918)
Supplement: Supplementary file 1 [file Table1.DOCX]

**Supplementary**

**TABLE 1** Two algorithms used for signal detection

| Algorithms | Equation | Criteria |
| --- | --- | --- |
| ROR | ROR=(a/c)/(b/d) | a≥3, 95%CI≥1 |
|  | 95%CI=e^ln(ROR)±1.96(1/a+1/b+1/c+1/d)^0.5^ |  |
| PRR | PRR=[a/(c+d)]/[c/(a+b)] | a≥3, PRR≥2, χ^2^≥4 |
|  | χ^2^=[(ad-bc)^2](a+b+c+d)/[(a+b)(c+d)(a+c)(b+d)] |  |

Equation: a, number of reports containing both the target drug and target adverse drug reaction; b, number of reports containing other adverse drug reaction of the target drug; c, number of reports containing the target adverse drug reaction of other drugs; d, number of reports containing other drugs and other adverse drug reactions. 95%CI, 95% confidence interval; χ^2^, chi-squared.

**TABLE 2** Daily dosage distribution of TAAs

|  | Fluconazole | Voriconazole | Posaconazole | Itraconazole | Isavuconazole |
| --- | --- | --- | --- | --- | --- |
| 50-200mg | 91 | 9 | 1 | 5 | 1 |
| 200mg | 76 | 28 |  | 32 |  |
| 200-300mg | 1 | 3 |  | 1 |  |
| 300mg | 8 | 14 | 23 | 1 |  |
| 400mg | 59 | 161 | 1 | 44 |  |
| >400mg | 15 | 31 | 18 | 10 | 1 |
| Unknown | 409 | 245 | 52 | 367 | 12 |
